# Quality of Sterile Male Tsetse after Long Distance Transport as Chilled, Irradiated Pupae

**DOI:** 10.1371/journal.pntd.0004229

**Published:** 2015-11-12

**Authors:** Momar Talla Seck, Soumaïla Pagabeleguem, Mireille D. Bassene, Assane Gueye Fall, Thérèse A. R. Diouf, Baba Sall, Marc J. B. Vreysen, Jean-Baptiste Rayaissé, Peter Takac, Issa Sidibé, Andrew G. Parker, Gratian N. Mutika, Jérémy Bouyer, Geoffrey Gimonneau

**Affiliations:** 1 Institut Sénégalais de Recherches Agricoles, Laboratoire National d’Elevage et de Recherches Vétérinaires, Service de Bio-écologie et Pathologies Parasitaires, Hann, Dakar, Sénégal; 2 Pan-African Tsetse and Trypanosomosis Eradication Campaign, Bobo-Dioulasso, Burkina Faso; 3 Centre de Coopération Internationale en Recherche Agronomique pour le Développement, Unité Mixte de Recherche Contrôle des Maladies Animales Exotiques et Emergentes, Campus International de Baillarguet, Montpellier, France; 4 Institut National de la Recherche Agronomique (INRA), Unité Mixte de Recherche 1309 ‘Contrôle des Maladies Animales Exotiques et Emergentes’, Montpellier, France; 5 Direction des Services Vétérinaires, Dakar, Sénégal; 6 Insect Pest Control Laboratory, Joint FAO/IAEA Programme of Nuclear Techniques in Food and Agriculture, International Atomic Energy Agency, Vienna, Austria; 7 Centre International de Recherche-Développement sur l’Élevage en Zone Subhumide, Bobo-Dioulasso, Burkina Faso; 8 Institute of Zoology, Section of Molecular and Applied Zoology, Slovak Academy of Sciences, Bratislava, Slovakia; 9 Centre de Coopération Internationale en Recherche Agronomique pour le Développement (CIRAD), Unité Mixte de Recherche ‘Interactions hôtes-vecteurs-parasites-environnement dans les maladies tropicales négligées dues aux trypanosomatides’, Montpellier, France; University of Perugia, ITALY

## Abstract

**Background:**

Tsetse flies transmit trypanosomes that cause human and African animal trypanosomosis, a debilitating disease of humans (sleeping sickness) and livestock (nagana). An area-wide integrated pest management campaign against *Glossina palpalis gambiensis* has been implemented in Senegal since 2010 that includes a sterile insect technique (SIT) component. The SIT can only be successful when the sterile males that are destined for release have a flight ability, survival and competitiveness that are as close as possible to that of their wild male counterparts.

**Methodology/Principal Findings:**

Tests were developed to assess the quality of *G*. *p*. *gambiensis* males that emerged from pupae that were produced and irradiated in Burkina Faso and Slovakia (irradiation done in Seibersdorf, Austria) and transported weekly under chilled conditions to Dakar, Senegal. For each consignment a sample of 50 pupae was used for a quality control test (QC group). To assess flight ability, the pupae were put in a cylinder filtering emerged flies that were able to escape the cylinder. The survival of these flyers was thereafter monitored under stress conditions (without feeding). Remaining pupae were emerged and released in the target area of the eradication programme (RF group). The following parameter values were obtained for the QC flies: average emergence rate more than 69%, median survival of 6 days, and average flight ability of more than 35%. The quality protocol was a good proxy of fly quality, explaining a large part of the variances of the examined parameters.

**Conclusions/Significance:**

The quality protocol described here will allow the accurate monitoring of the quality of shipped sterile male tsetse used in operational eradication programmes in the framework of the Pan-African Tsetse and Trypanosomosis Eradication Campaign.

## Author Summary

An area-wide integrated pest management campaign against *Glossina palpalis gambiensis* has been implemented in Senegal since 2010 that includes a sterile insect technique component. The sterile males used for the releases emerged from pupae that were produced and irradiated in Burkina Faso and Slovakia (irradiation done in Seibersdorf, Austria) and transported weekly under chilled conditions to Dakar, Senegal. Tests were developed to assess the quality (flight ability and survival) of sterile males. To assess flight ability, for each consignment a sample of 50 pupae (QC flies) was put in a cylinder filtering emerged flies that were able to escape the cylinder. The survival of these flyers was monitored under stress conditions. Remaining pupae (RF flies) were emerged and released in the target area of the eradication programme. The quality assessment of the QC flies was a good proxy of the quality of the RF flies. The quality protocol described here will allow the accurate monitoring of the quality of shipped sterile tsetse males used in operational eradication programmes.

## Introduction

Tsetse flies are hematophageous insects found in sub-Saharan Africa and are the main vectors of trypanosomes, the causative agents of African Animal Trypanosomosis (AAT) and Human African Trypanosomosis (HAT) [1]. The debilitating disease AAT limits the exploitation of fertile land for agricultural activities in an area of 10 million km^2^ [2] and is considered the main constraint to more productive livestock systems in sub-Saharan Africa [3,4]. The direct annual production losses of cattle in terms of decreased meat and milk production, abortions, etc. are estimated at USD 600–1200 million [5] and the overall annual losses in livestock and crop production have been estimated as high as USD 4750 million [6].

To suppress or eradicate these disease vectors, four methods that are environmentally and economically acceptable can be used in a context of area-wide integrated pest management (AW-IPM) approaches [4,7,8] i.e. the sequential aerosol technique (SAT) [9,10], the deployment of insecticide-impregnated traps/targets [11], the live-bait technology [12] and the sterile insect technique (SIT) [13,14]. The SIT is used throughout the world to suppress, eradicate, contain or prevent the introduction of several insect pests such as fruit flies [15], moths [16], screwworm flies [17–19], mosquitoes [20] and tsetse flies [14]. The effectiveness of the SIT to eradicate tsetse fly populations was demonstrated in Nigeria with *Glossina palpalis palpalis* [21], in Burkina Faso with *G*. *palpalis gambiensis*, *G*. *tachinoides* and *G*. *morsitans submorsitans* [13,22] and on Unguja Island, Zanzibar with *G*. *austeni* [14]. In Senegal, a programme is underway to eradicate a *G*. *p*. *gambiensis* population from the Niayes area [23–27]. This campaign is part of the Pan-African Tsetse and Trypanosomosis Eradication Campaign (PATTEC), an initiative of the African Heads of State and Government to ensure increased food security through better management of the tsetse fly and trypanosomosis problem [28].

The data of the feasibility study (2007–2010) indicated the potential to create a sustainable zone free of *G*. *p*. *gambiensis* in the Niayes [24,29], and therefore, the Government of Senegal opted for an AW-IPM approach that included an SIT component. An agreement was made between the Government of Senegal and the Centre International de Recherche-Développement sur l’Elevage en zone Subhumide (CIRDES) in Bobo-Dioulasso, Burkina Faso and the Slovak Academy of Sciences (SAS) in Bratislava, Slovakia to produce the sterile flies for the eradication campaign in Senegal. The male flies were transported as chilled pupae to Dakar where they could emerged under standard conditions [27].

In AW-IPM programmes that have an SIT component, the quality of the released sterile males remains one of the most crucial prerequisites for success, as flies of low quality (i.e. low survival rate and/or deformed wings) can’t compete with wild males in the field to mate with females and induce sterility in the native population [8,30,31]. Therefore, routine quality control procedures are crucial for the SIT component to identify weaknesses in production or handling procedures that result in low quality of the sterile males which may lead to potential failure of these programmes [32]. In past tsetse eradication campaigns [13,14,21,22] the rearing facility and target area were not far apart, so there was no need for pupal shipments. As an example, in the eradication programme on Unguja Island, Zanzibar, the sterile male flies were produced in Tanga, mainland Tanzania and the sterile adult flies were collected twice a week with light aircraft and released from the air in biodegradable cartons. A quality control system was implemented that consisted of taking one release carton before loading the aircraft in Tanga and one carton during the release flights. Both in Tanga and Unguja, the flies were released in a specially designed release arena and the following quality parameters assessed: number of flies in the box, mortality, number of non-flyers, sexing error, and feeding status [33].

In this study, we developed and validated a quality control protocol to assess the quality of male *G*. *p*. *gambiensis* that were irradiated and shipped as pupae under chilled conditions. Four biological parameters were measured: i) adult emergence, ii) percentage of flies with deformed wings, iii) flight ability of the sterile flies in the insectary and in the field and iv) survival of the flyers (those that were capable of flying out of the cylinder in the insectary) under stress conditions. These parameters were used to assess the reliability of this quality protocol to 1) predict field performance of the flies, 2) monitor and compare the performance of flies from two locations with different treatment protocols, and 3) develop quality criteria for use in feedback mechanisms to improve rearing systems.

## Methods

### Insectary

The study was carried out at the Institut Sénégalais de Recherches Agricoles / Laboratoire National de l’Elevage et de Recherches Vétérinaires, Service Bio-Ecologie et de Pathologies Parasitaires (ISRA/LNERV/BEPP) in Dakar. Insectary conditions were 24–25°C, 75 ± 5% RH and 12:12 light:dark photoperiod for emergence and the monitoring of the flies.

### Chilling and irradiation

Male *G*. *p*. *gambiensis* pupae from colonies kept at Burkina Faso and Slovakia were irradiated under chilled (4–6°C) conditions to lower their metabolic rate to prevent emergence [27,34]. The SAS pupae were irradiated in a Gammacell 220 (MDS Nordion, Ottawa, Canada) (dose rate of 3.11 Gy.sec^-1^ on 1 May 2012 and 2.19 Gy.sec^-1^ on 1 January 2015) or in an X-ray irradiator (Radsource 2400) (dose rate of 14.30 Gy.min^-1^) located at the FAO/IAEA Insect Pest Control Laboratory, Seibersdorf, Austria. The CIRDES pupae were irradiated in a ^137^Cs source for 24 minutes 30 seconds to give a dose of 110 Gy. The male pupae were packaged in cartons (for SAS) and in petri dishes (CIRDES) that were placed in insulated transport boxes containing phase change material packs (S8) (PCM Phase Change Material Products Limited, Cambridgeshire, United Kingdom) to maintain the temperature at 8–10°C and shipped to Dakar by commercial aircraft [27].

### Quality assessment of sterile males

The study was implemented from May 2012 to January 2015. A shipment of CIRDES and SAS pupae was received every week at the ISRA in Dakar. Each consignment contained two batches (1 and 2) of pupae that had a different larviposition date and consequently had been exposed to a different chilling period before shipping, i.e. batch 1 was chilled at 8°C for one day longer than batch 2 in the source insectary before transport. Each batch contained an average of 2500 pupae. A total of 50 pupae were sampled from each batch for the quality control test (QC) and the remaining pupae were emerged to be released in the operational eradication programme, i.e. the flies destined for release in the programme (RF). The pupae of the QC and RF groups were kept under the same environmental conditions (24–25°C, 75 ± 5% RH and a photoperiod of L:D 12:12 h). The 50 pupae for the QC group were selected to assess whether a small sample of each received pupae consignment was adequate to predict the quality of the shipped and released flies (RF).

### Pupae from the RF group

**Fig 1:**
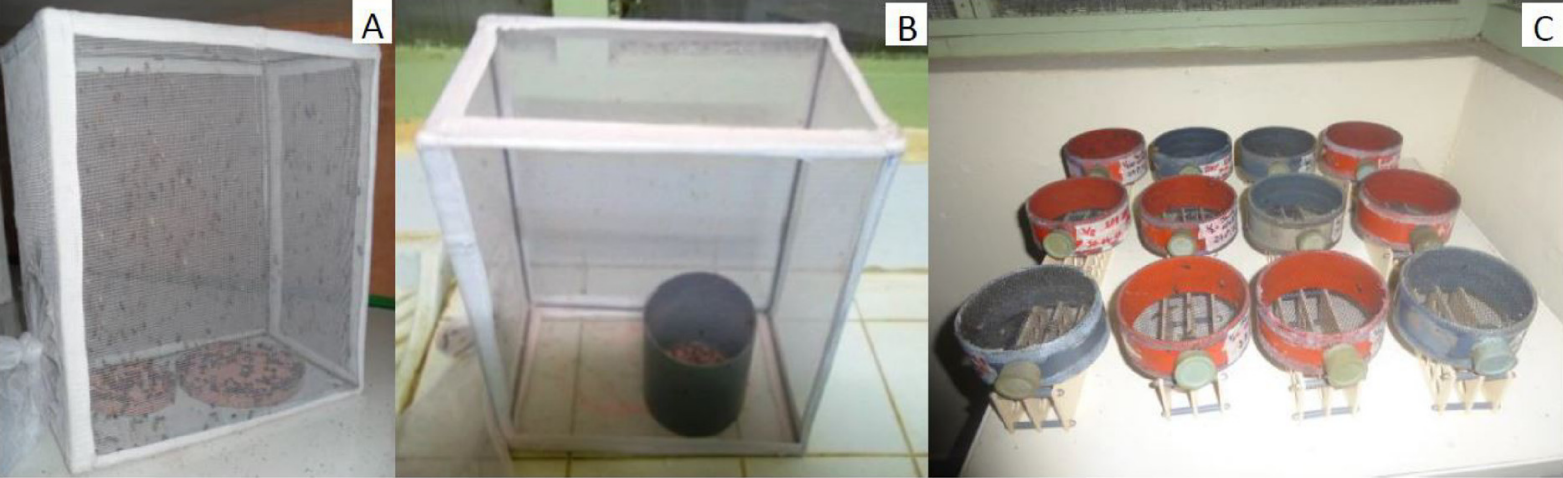
Illustration of the set up of quality assessment of sterile male flies during the tsetse eradication project in Senegal. (A) Emergence of flies destined for release (RF group), (B) emergence of quality control flies (QC group) from pupae placed in a flight cylinder, and (C) monitoring of the survival of QC flies without food. Every morning, flies that had escaped the cylinder were collected from emergence cages (B) and put into circular colony maintenance cages for assessing their survival (1 cage per day).

Pupae from the RF group were placed in Petri dishes under ~1cm of sand mixed with a fluorescent dye (DayGlo) (0.5g dye/200g of sand), to mimic the natural emergence conditions in the soil (Fig 1A) and to allow discrimination from wild flies in the monitoring traps as these sterile male flies were released in the operational programme. Emerged flies were sorted and classed as “normal” (flies with no apparent morphological deficiencies) and “abnormal flies” (i.e. with deformed wings). Normal flies were offered at least three bovine blood meals (originating from slaughterhouse of Dakar, with the consent from the slaughterhouse to obtain the blood samples from livestock) containing 10 mg of the trypanocidal drug isometamidium per litre of blood using the *in vitro* silicon membrane feeding system before being transported to the field for release. The trypanocidal drug prevents the cyclical development of trypanosomes in the released sterile males [35–37]. Irradiated and marked males were transported by car to the release sites (~ 1 hour for Diacksao Peulh and Kayar and 10 minutes for the Parc de Hann [24]) in Roubaud-type cages (4.5 x 13 x 8 cm) that were covered with netting with a mesh size of 1 mm x 1 mm, each containing on average 120 sterile males. Cages were kept in climate controlled containers (temperature and humidity of 24–26°C, 75 ± 5% respectively) during the transport and temperature and humidity were recorded every 5 minutes with a Hobo data logger. Flies were released every Friday afternoon between 16:00 and 18:00 h over a white cloth (2 x 1.5 m). Males remaining on the cloth after 5 minutes were counted and considered as non-flyers. Ground releases of these flies took place from May 2012 to March 2013. Thereafter, all sterile male flies were released by air.

### Pupae of the QC group

The pupae of the QC group were kept under the same conditions as the RF group but the Petri dishes with the pupae were put in a flight cylinder, i.e. a PVC tube 10 cm high and 8.4 cm in diameter (Fig 1B). The inner wall of the cylinder was coated with unscented talcum powder to prevent the flies from crawling out. This method was initially developed for routine quality assessments of sterile fruit flies [38,39] and moths [40], and adapted here to tsetse flies. This protocol gave an indication of the propensity of the sterile male flies to fly out of the cylinder and only those flies that managed to escape the flight cylinder after emergence were considered as “available for the SIT”. Flies with deformed wings and those with normal wings but unable to escape the flight tube were counted, as well as the number of pupae that did not emerge.

### Survival under stress at the insectary

The survival of the sterile males of the QC group that escaped the flight cylinder was assessed under stress conditions (no food). Every morning (except Sundays), the emerged flies were collected and transferred to standard fly holding (10.3 cm diameter and 4.5 cm high) cages (Fig 1C). The flies emerged on a given day were pooled in one cage. Dead flies were counted daily and removed from the cages.

### Data analysis

The data sets (both QC and RF groups) on percentage emergence, flies with deformed wings and flight ability were each divided into training and test sets. The training set was used to build the model and the test set to measure its performance [41]. For the data on emergence and percentage of flies with deformed wings, 60% of the entire data set (n = 364 rows), selected at random, was used as a training set and the remaining as the test set. For the flight ability, 75% of the entire data set (n = 80 rows) was used for the training set and 25% for the test set. The difference in the proportion of data used for the training set in the first and second cases was related to sample size.

A binomial linear mixed effect model was used to analyze emergence rates. The emergence rate measured within the QC group, the origin of the pupae (CIRDES and SAS), the batches (1 and 2) and their second and third order interactions were used as explanatory variables and the emergence rate of the RF group as the response variable. The shipment date was considered as a random effect. The best model was selected on the basis of the lowest corrected Akaike information criterion (AICc), and the significance of fixed effects was tested using the likelyhood ratio test [42,43]. The R^2^ (coefficient of determination) was used to describe the proportion of variance explained by the model for the training and test data sets [44,45].

The same analysis was used for the percentage of flies with deformed wings and the percentage of flyers.

Flight ability was analyzed between QC and RF groups using only the CIRDES data sets because field data were not available for the SAS shipments. Flight ability was compared among years (2012, 2013 and 2014) using the same binomial model.

The survival of the sterile males of the QC group that had escaped from the flight cylinder and kept under starvation was analyzed using Kaplan-Meier survival curves. Survival curves were compared between origins (CIRDES and SAS), batches (1 and 2) and years using the coxph model [46]. The median survival was considered to be the average probable survival of the studied flies. The R Software (version 3.1.0) was used for all statistical analyzes [47].

### Data accessibility

The complete data sets are available in S1 and S2 and S3 Tables.

### Ethical statement

The study was conducted in the framework of the tsetse eradication campaign in Senegal, led by the Directorate of Veterinary Services, Ministry of Livestock and the ISRA/LNERV, Ministry of Agriculture and Rural Equipment. This project received official approval from the Ministry of Environment of Senegal, under the permit N°0874/MEPN/DE/DEIE/mbf.

## Results

### Validation of the protocol to assess fly quality

A total of 1,581,366 irradiated pupae were used for this study of which 1,271,121 (80.4%) originated from the CIRDES insectary (123 shipments) and 310,245 (19.6%) pupae originated from the SAS insectary (53 shipments).

The emergence rate of pupae of the RF group was significantly greater for shipments originating from CIRDES than those from SAS (*P* < 10^−3^; Table 1), as well as for batch 2 pupae than batch 1 pupae regardless of the origin of pupae (*P* < 10^−3^; Table 1). The percentage of flies with deformed wings was significantly lower for the flies that originated from the CIRDES than the SAS flies and for batch 2 pupae than batch 1 pupae regardless of the origin (*P* < 10^−3^; Table 1). The flight ability of the CIRDES flies in the field was significantly better for flies derived from batch 2 pupae than batch 1 pupae (*P* < 10^−3^; Table 1).

**Table 1:** Average values ±sd (%) of different parameters from the QC and RF presented by origin and batch.

	CIRDES		SAS	
Batch	1	2	1	2
	Emergence			
RF	72.7±13.6^a^	78.2±11.5^b^	55.9±18.4^c^	66.4±16.3^d^
QC	71.1±15.3^a^	76.4±13.7^b^	68.9±19.0^a^	74.5±16.6^b^
	Flies with deformed wings			
RF	8.6±3.6^a^	7.3±4.5^b^	14.2±8.0^c^	12.0±7.5^d^
QC	12.4±7.1^d^	11.6±6.8^d^	9.8±7.5^a^	7.8±6.3^b^
	Flight ability			
RF	54.1±14.6^a^	56.0±12.6^b^	-	-
QC	34.1±17.8^c^	35.8±18.0^c^	-	-

RF = Pupae destined for release in the operational programme

QC = Pupae of the quality control test

For each parameter (emergence, flies with deformed wing and flight ability) the values with the same letters (amongst columns and rows) are not significantly different (P > 0.05).

Adult emergence of pupae of the QC group was similar between origins (*P* = 0.8) but differed between batches regardless of the origin (*P* < 10^−3^; Table 1). The percentage of flies with deformed wings that emerged from the SAS pupae was significantly lower than that for the CIRDES pupae (*P* < 10^−3^; Table 1). It was similar between batches for CIRDES and different for SAS (*P* < 10^−3^; Table 1). The flight ability was similar between batches (*P* > 0.05; Table 1).

**Fig 2:**
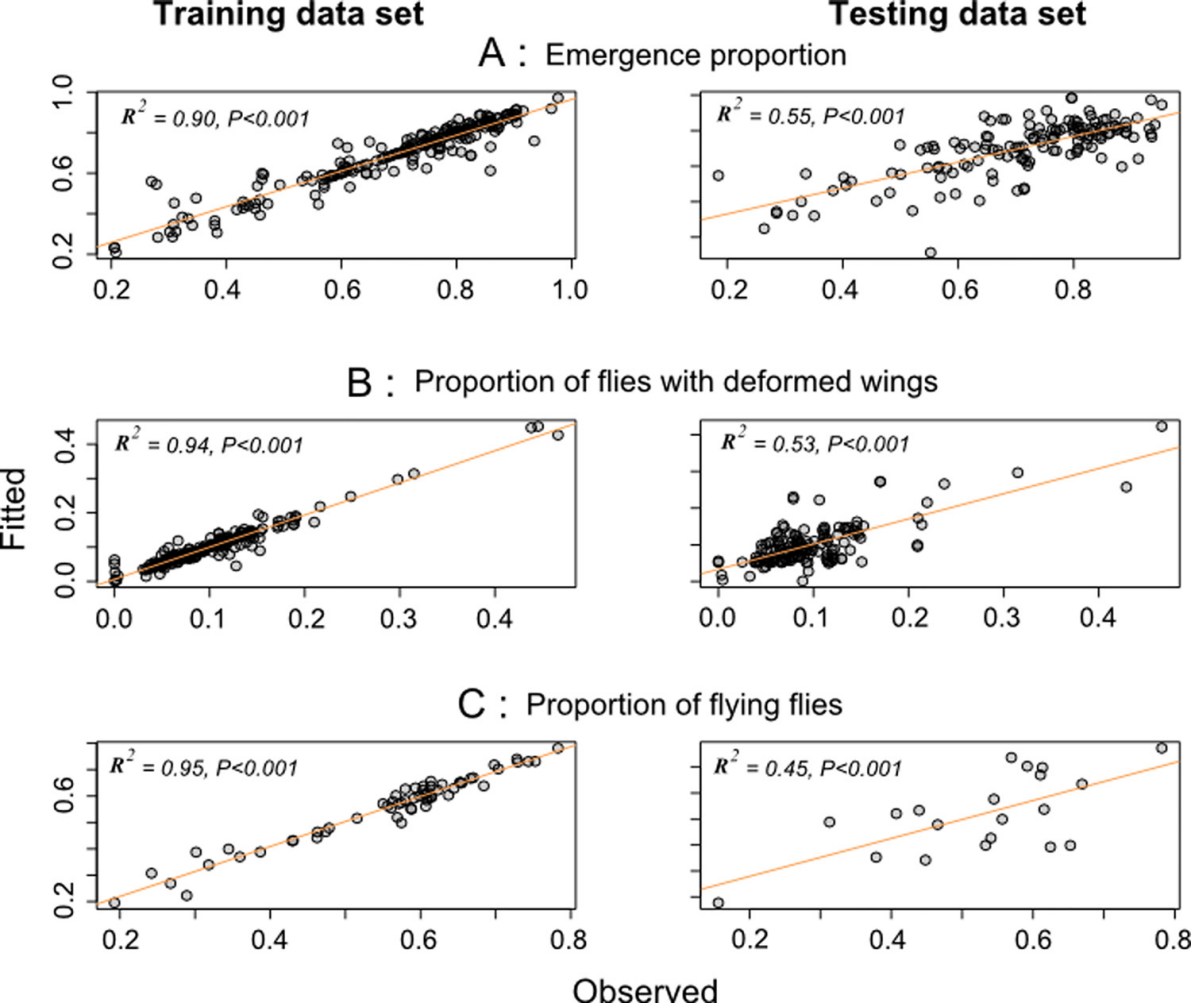
Relationship between the fitted and observed values of the quality assessment parameters. (A) Emergence of pupae, (B) flies with deformed wings, and (C) flying flies from training and test sets of the QC and RF flies. The training set of each parameter (60% of emergence and flies with deformed wings data and 75% of flight ability data) was used to build the model and the test set (the rest of data) to measure the model performance. The fitted values were obtained from the binomial linear mixed model. The emergence rate, percentage of flies with deformed wings, percentage of flying flies within the QC group, their origins and batches were used as explanatory variables and the RF group parameters as explained variables with the shipment date as a random factor. The orange lines give the regressions.

The comparison of the different parameters between the QC and the RF groups showed that the emergence rates were similar for the CIRDES flies while they were significantly greater in the QC group of the SAS flies (*P* < 10^−3^; Table 1). The percentage of flies with deformed wings was lower in the RF group as compared with the QC group for the CIRDES pupae, whereas it was the opposite for the SAS pupae (*P* < 10^−3^; Table 1). The percentage of the CIRDES flies escaping the flight cylinder in the insectary was significantly lower than the percentage of flies taking off in the field after the release (*P* < 10^−3^) i.e. 34.9 ± 17.8% and 55.1 ± 13.5%, respectively. The flight ability of batch 2 flies of the RF group was significantly greater than for batch 1 flies (*P* < 10^−3^) whereas no difference was observed between batches of the QC group (*P* = 0.6; Table 1). The predicted probabilities of occurrence using the QC data allowed us to predict the results observed in the RF group with good accuracy: the emergence rates, percentage of flies with deformed wings and flight ability were strongly correlated to predictions of the training data set (*P* < 10^−3^; R^2^ of 0.90, 0.94 and 0.95 respectively; Fig 2). For the test data set, the model predicted 55%, 53% and 45% of the variances respectively (*P* < 10^−3^, Fig 2).

### Survival under stress

**Fig 3:**
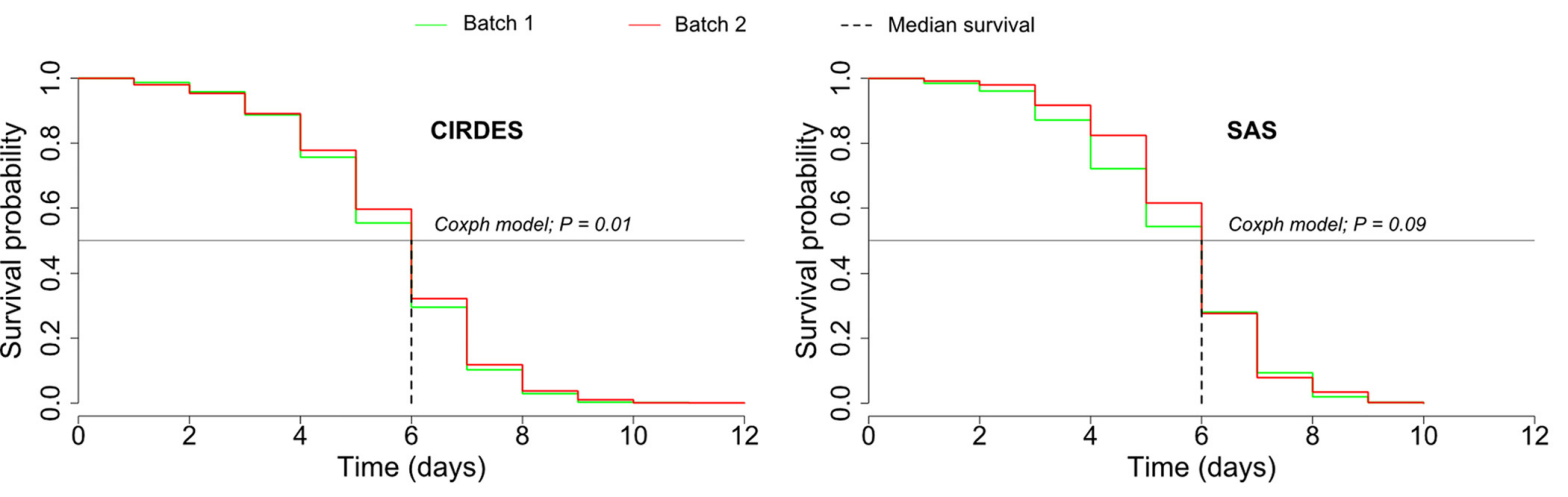
Survival curves of sterile males that were kept without food and had to survive on their fat reserves. Only flies able to escape the flight cylinder were used for this study. The horizontal black line (Y axis = 0.5) was used to determine the median survival (dotted lines).

Survival curves of QC flies kept under starvation are presented by batch and origin in Fig 3. Flies from batch 2 pupae survived significantly longer than those from batch 1 pupae for CIRDES (*P* = 0.01) whereas for SAS, batch 2 flies survived marginally longer than from batch 1 (*P* = 0.09) shipments. The CIRDES flies survived marginally longer than the SAS flies (*P* = 0.06). The median survival was 6 days regardless of the batch and origin of pupae (Fig 3). The maximum survival observed was 12 days after emergence for the CIRDES (batch 2) and 10 days for the SAS (batch 2) flies.

### Temporal variation of male quality

From 2012 to 2014, the percentage of QC flies escaping the cylinder gradually increased regardless of the origin of pupae (*P* < 10^−3^; Table 2). Flies lived significantly longer in the survival tests in 2013 and 2014 as compared with 2012 for both CIRDES and SAS flies (*P* < 10^−3^). Thus, the quality of sterile male flies (flight ability and survival) was significantly improved among years and these improvements were more prominent for flies from SAS (Table 2).

**Table 2:** Percentage (±sd) of flies escaping the flight cylinder and their median survival by origin and year.

Origin of pupae	% flies escaping the tube			Median survival (days)		
	2012	2013	2014	2012	2013	2014
CIRDES	34.9±16.9^a^	39.7±19.3^a^	48.0±19.0^b^	5^a^	6^b^	6^b^
SAS	14.0±17.0^a^	24.2±10.3 ^a^	54.1±27.8^b^	3^a^	6^b^	6^b^

The data on the same line with a letter in common are not significantly different (P > 0.05).

## Discussion

The quality protocol implemented in this study was developed for a programme that required long distance transport of chilled male tsetse pupae and was shown to be a good proxy for the insectary rearing output. Indeed, the emergence rates, percentage of flies with deformed wings and flyers from the QC and RF groups were highly correlated. Overall, these results highlight that the quality protocol procedures had no negative impact on adult emergence and predicted well the amount of sterile males available for the SIT component. In eradication programmes such as the one implemented in the Niayes of Senegal, thousands of sterile male flies need to be processed weekly for release requiring many preliminary activities in the insectary (to separate and to count normal and abnormal flies after emergence, assess mortality rate and percentage of non-flyers after release in the field). With the results obtained from the QC group, it was shown that all these parameters predicted well the biological quality of the sterile male flies, which will reduce considerably the work load. More importantly, multiple handling of flies (generally at 2–4°C for the sorting) generates stress which reduces their quality which can be avoided using a sample for the quality control test [27].

Quality control protocols for SIT programs were initially developed for fruit flies, especially the Mediterranean fruit fly *Ceratitis capitata* and has more recently been extended to *Anastrepha* and *Bactrocera* fruit fly species [38,39]. For these insects, the average flight ability after irradiation and transport of pupae was 65% for *C*. *capitata*, 75% for *Anastrepha suspensa* and 55% for *Bactrocera oleae* [38,39,48]. The flight ability obtained in the present study with *G*. *p*. *gambiensis*, BKF strain was on average 35.8 ± 18.4%. Although caution is required when comparing data from different species and when pupae were shipped under different conditions, it provides an indication that our results with *G*. *p*. *gambiensis* were rather low. This low propensity to fly could be due to mechanical shocks and vibrations that were absorbed by the pupae during transport or possibly different handling procedures in the different insectaries. In addition, the length of the cooling period of the pupae seems to be an important quality reducing factor, especially in terms of emergence rate. The impact of these different variables on emergence of adults was shown before [27]. Adults emergence may also be affected by excessive temperatures or inappropriate relative humidity during the rearing process [30]. In addition, it is well established that irradiation could potentially lower the quality of the produced flies especially when the irradiation dose that is required to obtain 95–100% sterility is high and therefore results in severe somatic damage [30,49–51].

The released sterile males must be active to find a blood meal, shelter and to compete with wild males for mating with wild females and successfully transfer the sterile sperm, and they must survive long enough to be able to find the virgin females [30]. Data of the QC group indicated that about 20% of the flies that emerged were “normal-looking” flies that had their wings deployed but did not escape the flight cylinder. What is measured here is the propensity of the flies to fly i.e. some of those flies that stayed in the cylinder probably can fly, but for one or the other reason they don’t. This was confirmed by the data from the field in that most of these flies were able to take off from the release cloth; however, they still might be poor flyers (but this was not assessed in this study). Indeed, after the preliminary sorting at the insectary, all normal-looking flies (i.e those that were mobile and had deployed wings) were transported to the field and released using the ground release protocol where the flies were released on a cloth (2 x 1.5 m) and checked after 5 minutes.

These observations confirm the necessity to implement a quality control protocol for sterile males to make eradication campaigns more effective. Weekly data on the percentage of released sterile males as compared to the number of shipped pupae allows for crucial feedback information to the rearing facility and to better plan the operational phase of the SIT component of AW-IPM programmes [4]. For example, by improving the packaging and transport protocols (such as the use of cotton for the CIRDES pupae and sawdust/vermiculite for the SAS pupae to cushion the mechanical shocks) flight ability was increased significantly reaching 55% in 2014.

There was no difference between the survival of the sterile males that emerged from the CIRDES and the SAS pupae. This indicates that the quality of the blood diet and the performance of the females in the colonies of the two rearing facilities were equivalent. The sterile males did not receive any blood meals during the survival experiment, and hence, their survival depended only on the fat reserves acquired during larval development. As tsetse reproduce by adenotrophic viviparity [4], these fat reserves are closely linked to the quality of the blood meals that are taken by the female parents. Under these conditions, the median survival of sterile males was 6 days regardless of the origin of the pupae with more than 80% and 10% surviving until 4 and 8 days after emergence, respectively. These results were similar to thoses observed with *G*. *pallidipes*, i.e. 90% of *G*. *pallidipes* males that emerged from pupae that had been exposed to a low temperature of 15°C survived unfed until 4 days but less than 10% survived after 8 days [52]. In order to simulate the proposed use of the chilled adult release system for area-wide tsetse SIT, the tenerale male flies of the same tsetse species exposed to a temperature of 7°C for 48 and 72 hours followed by 6 hours at 4°C and monitored without being offered a blood meal showed a median survival of 4 days [52].

The flies that emerged from batch 2 pupae survived on average longer than those emerging from batch 1 pupae indicating that the duration of the chilling at 8°C had a negative impact on fly quality. The median survival of the sterile males of 6 days without food as observed under laboratory conditions is encouraging, as the sterile males that are destined for release are being offered a blood meal at least three times before being released. This will undoubtedly increase their fat reserves thus enhancing their survival until they have found a host and hence, their competitiveness.

In conclusion, although the quality protocol data indicated that the percentage of flyers was less than 40%, the quality of the transported sterile pupae improved with time. More importantly, the data from the field indicate that the competitiveness of those male flies that were released was very good [53] resulting in excellent progress in the eradication campaign [54–56]. Research continues to improve the transport conditions of the pupae to potentially further increase the proportion of flyers.

## Supporting Information

S1 Table Database for the emergence parameters of irradiated pupae after long distance transport as chilled pupae. (CSV)

S2 Table Database for the flight ability of the CIRDES flies. (CSV)

S3 Table Database for the survival of the sterile males that escaped the flight cylinder. (CSV)
